# A Novel Role of Protecadherin-7 in Regulation of Pydc3 Expression and the IFN-β Response During Osteoclast Differentiation

**DOI:** 10.3390/cells14161298

**Published:** 2025-08-21

**Authors:** Hyunsoo Kim, Noriko Takegahara, Yongwon Choi

**Affiliations:** Department of Pathology and Laboratory Medicine, Perelman School of Medicine, University of Pennsylvania, Philadelphia, PA 19104, USA; hyunsoo3@pennmedicine.upenn.edu (H.K.); tnoriko@pennmedicine.upenn.edu (N.T.)

**Keywords:** Pcdh7, Pydc3, IFN-β, osteoclast differentiation

## Abstract

Protocadherin-7 (Pcdh7) is a member of the protocadherin family, a subgroup of the cadherin superfamily. We previously demonstrated that Pcdh7 functions as a signaling receptor in osteoclast differentiation. In this study, we investigated the potential gene regulatory role of Pcdh7 in this process and identified Pyrin domain-containing protein 3 (Pydc3) as a key mediator of Pcdh7-mediated regulation of osteoclast differentiation. Differential gene expression analysis comparing wild-type (Pcdh7^+/+^) and Pcdh7-deficient (Pcdh7^−/−^) cells revealed a significant upregulation of *Pydc3* in Pcdh7^−/−^ cells. RNAi-mediated knockdown of *Pydc3* rescued the impaired osteoclast differentiation in Pcdh7^−/−^ cells, whereas overexpression of *Pydc3* suppressed osteoclast differentiation in Pcdh7^+/+^ cells, suggesting that Pydc3 negatively regulates osteoclast differentiation. Additionally, Pcdh7^−/−^ cells showed elevated expression of interferon response genes and increased production of interferon-β (IFN-β). Neutralization of IFN-β signaling using anti-IFN-β and/or anti-interferon alpha and beta receptor 1 (IFNAR1) antibodies significantly restored osteoclast differentiation in Pcdh7^−/−^ cells. Collectively, these findings uncover a novel role for Pcdh7 in osteoclast differentiation through regulation of *Pydc3* expression and IFN-β production.

## 1. Introduction

Osteoclasts are large, multinucleated cells specialized for bone resorption. They originate from the hematopoietic lineage and differentiate from myeloid precursors [1,2]. Protocadherin-7 (Pcdh7) is a member of the protocadherin subgroup within the cadherin superfamily, a group of calcium-dependent cell adhesion proteins [3]. We previously demonstrated that Pcdh7 is essential for osteoclast differentiation, functioning as a signaling receptor. During RANKL-induced osteoclast differentiation, Pcdh7 ligation activates protein phosphatase 2 A (PP2A), which subsequently activates glycogen synthase kinase-3b (GSK3β). GSK3β then activates small GTPases, including RhoA, promoting osteoclast differentiation and multinucleation [2,4]. However, whether Pcdh7 also contributes to gene regulation during osteoclast differentiation remains unknown.

The type I interferon (IFN) family consists of pleiotropic cytokines that regulate cell-intrinsic immune responses, with IFN-α and IFN-β being the most extensively studied [5,6]. These cytokines signal through a shared heterodimeric receptor composed of interferon alpha and beta receptor 1 (IFNAR1) and interferon alpha and beta receptor 2 (IFNAR2) subunits, leading to the induction of interferon-stimulated genes (ISGs). In the context of bone metabolism, the role of type I IFNs has been well characterized [7]. Upon stimulation with the osteoclast differentiation factor receptor activator of nuclear factor-kB ligand (RANKL), osteoclast precursors transiently produce low levels of IFN-β [8]. IFN-β inhibits the expression of c-Fos, a key transcription factor essential for osteoclast differentiation, thereby suppressing the differentiation process [8]. Consistently, genetic deficiency of either IFNAR1 or IFN-β results in enhanced osteoclast formation and reduced bone mass in mice [8,9]. These findings underscore the role of Type I IFN signaling as a negative feedback mechanism that regulates RANKL signaling and maintains osteoclast-mediated bone homeostasis.

Pyrin domain-only proteins (POPs) are a family of inflammasome regulators [10,11]. Three POPs—POP1, POP2, and POP3—have been identified in several species, including humans, but are absent in mice. These proteins regulate inflammasome assembly by binding to and sequestering the pyrin domain (PYD) in apoptosis-associated speck-like protein containing a CARD (ASC), a key adaptor required for the activation of downstream effectors such as caspase-1. POPs also bind to PYD-containing pattern recognition receptors, thereby preventing inflammasome formation. Among them, POP3 is particularly responsive to IFN-β signaling [12]. It functions as an inhibitor of AIM2-like receptors (ALRs) inflammasome formation and promotes a type I interferon response. Pyrin domain-containing protein 3 (Pydc3), also known as interferon activated gene 208 (Ifi208), is considered a putative functional analog of human POP3 in mice [13]. Pydc3 has been shown to be upregulated by IFN-β in mouse CD11b^+^ macrophages and has been identified as a target of PGD_2_/DP1 signaling in virus-induced central nervous system inflammation [14].

In this study, we identified a specific role for Pcdh7 in regulating *Pydc3* expression and the IFN-β response during osteoclast differentiation. Transcriptomic analysis revealed significant upregulation of *Pydc3* and ISGs in Pcdh7^−/−^ cells. Through RNAi-mediated gene silencing and overexpression approaches, we demonstrated that *Pydc3* negatively regulates osteoclast differentiation. Pcdh7 deficiency resulted in abnormal IFN-β production, and neutralization of IFN-β signaling restored the impaired osteoclast differentiation observed in Pcdh7^−/−^ cells. Together, these findings uncover a previously unrecognized function of Pcdh7 during osteoclast differentiation.

## 2. Materials and Methods

### 2.1. RNA Sequencing

Total RNA was extracted from the cultured cells using the RNeasy Plus Mini Kit (QIAGEN) according to the manufacturer’s protocol. The purified RNA samples were carefully packed on dry ice and shipped to Azenta Life Sciences for further processing. At Azenta, cDNA library preparation, RNA sequencing, and comprehensive data analysis were performed. The Standard RNA sequencing service was employed, enabling the construction of libraries suitable for both coding and long non-coding RNA profiling. mRNA was isolated using poly (A) selection. Sequencing was conducted on the Illumina platform. Sequence reads were trimmed to remove possible adapter sequences and mapped to the Mus musculus GRCm38 reference genome available on ENSEMBL. Unique gene hit counts were calculated. Gene hit counts were extracted from the sequencing data and analyzed to assess differences in expression between Pcdh7^+/+^ and Pcdh7^−/−^ cell culture groups using the DESeq2 package. Statistical analysis was performed with the Wald test, which was used to calculate *p*-values and log2 fold changes for each gene. Genes meeting the criteria of an adjusted *p*-value less than 0.05 and an absolute log2 fold change greater than 1 were classified as differentially expressed. This approach ensured that only statistically significant and biologically relevant changes in gene expression were considered. The experiment was conducted with three biological replicates.

### 2.2. Osteoclast Differentiation

Osteoclast differentiation was performed based on a well-established and previously described protocol [15]. Initially, bone marrow cells were harvested and cultured under specific conditions to generate bone marrow-derived macrophages (BMMs). This was achieved by incubating the bone marrow cells in a culture medium supplemented with macrophage colony-stimulating factor (M-CSF) at a concentration of 60 ng/mL for a duration of three days. This step promotes the proliferation and survival of macrophage precursor cells. After the initial culture period, the resulting BMMs were carefully seeded into 96-well culture plates at a consistent density of 1 × 10^4^ cells per well, then briefly centrifuged at 200× *g* for 3 min to promote uniform cell attachment and facilitate subsequent growth and differentiation. The cells were then cultured for an additional three days in the presence of both M-CSF (60 ng/mL) and receptor activator of nuclear factor κB ligand (RANKL) at 150 ng/mL. The combined presence of these two factors is essential, as M-CSF supports macrophage viability and proliferation, while RANKL drives the differentiation process toward mature osteoclasts. Following the culture period, the formation of differentiated osteoclasts was confirmed by staining and visualization using the Leukocyte Acid Phosphatase Kit (Sigma-Aldrich, St. Louis, MO, USA; Cat.#387A-1KT). This kit specifically detects acid phosphatase activity, a hallmark enzymatic function of osteoclasts. The staining procedure was carried out strictly in accordance with the manufacturer’s guidelines to ensure accurate identification and assessment of osteoclast differentiation. BMMs and osteoclasts were used for the following experiments. Experiments were conducted at least three times.

### 2.3. Retroviral Transduction

For Pydc3 silencing, retroviral particles were produced using Plat-E packaging cells transfected with pSuper vectors encoding shRNAs targeting Pydc3 (5′-GCCTAAGTTTCCATTACTTTC-3′). For Pydc3 overexpression, mouse Pydc3 cDNA (OriGene technology, Rockville, MD, USA Cat.#MR217014) was subcloned into the pMX vector. Empty pSuper or pMX vectors were used as negative controls. Retroviral supernatants were collected, filtered through a 0.45 μm syringe filter, and used to transduce BMMs overnight in the presence of hexadimethrine bromide (6–8 μg/mL) and M-CSF (120 ng/mL).

### 2.4. Immunoblotting

Cells were subjected to lysis using RIPA lysis and extraction buffer (Thermo Scientific™, Waltham, MA, USA, Cat.#89901), which was supplemented with a cocktail of protease and phosphatase inhibitors (Roche, Indianapolis, IN, USA). The lysates were quantified and equal amounts of proteins from samples were separated by electrophoresis on a 4–15% SDS-polyacrylamide gradient gel. Following electrophoretic separation, the proteins were transferred onto a polyvinylidene difluoride (PVDF) membrane, and the membrane was then incubated with specific primary antibodies. For protein visualization, the blots were scanned and analyzed using the LI-COR Odyssey Fc Imager (LI-COR Biosciences, Lincoln, NE, USA). The primary antibodies employed in this study included anti-Flag M2 antibody (Sigma Aldrich, St. Louis, MO, USA; Cat.#A8592), used for detecting Flag-tagged proteins, and anti-Actin antibody (Santacruz Biotechnology, SantaCruz, CA, USA, Cat.#SC-47778), serving as a loading control to ensure equal protein loading across samples.

### 2.5. Quantitative PCR

Total RNA was extracted from cells following two to three washes with Dulbecco’s phosphate-buffered saline (DPBS) to remove residual medium and debris. Cells were lysed in 1 mL of TRIzol^®^ reagent (Invitrogen, Carlsbad, CA, USA) according to the manufacturer’s protocol. From the isolated RNA, 1–5 μg of total RNA was reverse-transcribed into complementary DNA (cDNA) using random hexamer primers and SuperScript™ III reverse transcriptase (Invitrogen, Carlsbad, CA, USA). The resulting cDNA, corresponding to 10 ng of input total RNA, was subjected to quantitative PCR (qPCR) analysis using the QuantStudio™ 3 Real-Time PCR System (Thermo Fisher Scientific, Waltham, MA, USA) and the following specific TaqMan probes: Pydc3 (Mm04206759_mH), Ifnb (Mm00439552_s1), and 18S gene (Hs99999901_s1) was used as the internal control, and the relative expression level were quantified using the comparative CT (cycle threshold) method.

### 2.6. ELISA

To determine IFN-β concentrations, culture supernatants were harvested from treated cells and passed through a 0.2 μm syringe filter to eliminate cellular debris. The clarified samples were subsequently analyzed using a mouse IFN-β ELISA kit (Abcam, Cambridge, MA, USA. Cat. #ab252363), following the protocol provided by the manufacturer. Each standard and sample was assayed in duplicate to ensure measurement accuracy. Absorbance values were recorded at 405 nm using an EMax Plus microplate reader (Molecular Devices, Sunnyvale, CA, USA), and cytokine concentrations were calculated from the corresponding standard curve.

### 2.7. RNA Extraction from Bone Tissue

Bone samples were collected aseptically into sterile containers containing phosphate-buffered saline (PBS). Approximately 160 mg of bone tissue was transferred to a sterile 10 cm Petri dish using sterile forceps. TRIzol^®^ reagent (1 mL) was added to the tissue, which was subsequently minced into a coarse slurry using sterile scissors. Homogenization was completed using a Dounce tissue grinder until no visible fragments remained. The homogenate was transferred to a 1.5 mL microcentrifuge tube and placed on ice for 5 min. Samples were centrifuged at 12,000× *g* for 10 min at 4 °C, and the supernatant was collected in a fresh 1.5 mL microcentrifuge tube. To shear genomic DNA and reduce lysate viscosity, the supernatant was passed slowly through a 21-gauge needle at least 6 times. Chloroform (0.15 volumes) was added, and the tube was shaken vigorously for 15 s. Samples were centrifuged at 10,000× *g* for 15 min at 4 °C, and the upper aqueous phase was carefully transferred to a new 1.5 mL tube. An equal volume of isopropanol was added to the aqueous phase, followed by gentle inversion (5–6 times) to mix. Samples were incubated at room temperature for 10 min to precipitate RNA, then centrifuged at 10,000× *g* for 10 min at 4 °C. The supernatant was discarded, and the RNA pellet was washed with 1 mL of ice-cold 75% ethanol. Following centrifugation at 10,000× *g* for 5 min at 4 °C, the supernatant was removed, and tubes were briefly centrifuged again for ~15 s to remove residual ethanol. The RNA pellet was air-dried for 3 min, ensuring it was not over-dried, and subsequently dissolved in 30–80 μL of nuclease-free distilled water. RNA concentration and purity were measured with a NanoDrop spectrophotometer. The RNA was then aliquoted and stored at −70 °C or lower until further use.

### 2.8. Statistics

One-way ANOVA, two-way ANOVA, or 2-tailed paired Student’s *t* test were used for comparisons involving three or more independent groups. A *p* value of less than 0.05 was considered statistically significant. Statistical analyses were performed using GraphPad Prism software (version 10).

## 3. Results

### 3.1. Identification of Pydc3 as a Pcdh7-Regulated Gene

**Figure 1:**
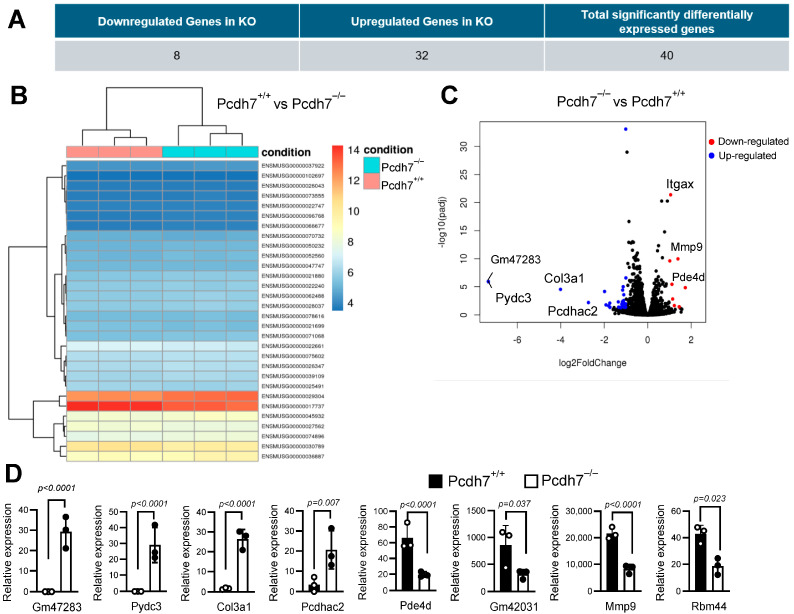
Pydc3 is highly expressed in Pcdh7^−/−^ cells. (**A**) Eight genes were identified as significantly downregulated in Pcdh7^−/−^ cells, and 32 genes were identified as significantly upregulated in Pcdh7^−/−^ cells, totaling 40 significantly expressed genes. (**B**) Heat map of differentially expressed genes between Pcdh7^−/−^ and Pcdh7^+/+^ cells. (**C**) Volcano plot of differentially expressed genes between Pcdh7^−/−^ and Pcdh7^+/+^ cells. Significantly expressed genes were marked in blue (up-regulated in Pcdh7^−/−^ cells) and red (down-regulated in Pcdh7^−/−^ cells). (**D**) Graphs of expression values of representative differentially expressed genes between Pcdh7^+/+^ and Pcdh7^−/−^ cells. Each dot represents a biological replicate.

To identify potential target genes regulated by Pcdh7 during osteoclast differentiation, we performed differential gene expression analysis comparing Pcdh7^+/+^ and Pcdh7^−/−^ cells. Bone marrow cells from Pcdh7^+/+^ and Pcdh7^−/−^ mice were cultured with M-CSF for three days to generate bone marrow-derived monocytes (BMMs), followed by an additional day of culture with RANKL to induce preosteoclasts, during which Pcdh7 expression peaked [16]. Total RNA was isolated from preosteoclasts and subjected to RNA sequencing and differential gene expression analysis (Figure 1). The analysis identified 40 significantly differentially expressed genes: 8 genes were downregulated and 32 were upregulated in Pcdh7^−/−^ cells (Supplementary Table S1). Among these, Gm47283 and Pydc3 were exclusively detected in Pcdh7^−/−^ cells. While Gm47283 encodes a long non-coding RNA, Pydc3 is a protein-coding gene. We therefore focused our subsequent investigations on Pydc3 to explore its role in Pcdh7-mediated regulation of osteoclast differentiation.

### 3.2. Pydc3 Negatively Regulates Osteoclast Differentiation

**Figure 2:**
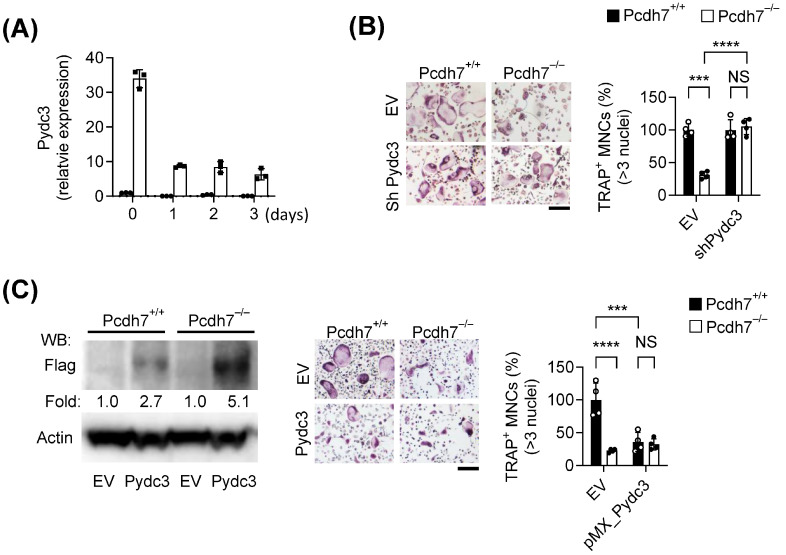
Pydc3 negatively regulates osteoclast differentiation. (**A**) A Representative expression profile of *Pydc3* in Pcdh7^+/+^ (circle) and Pcdh7^−/−^ (square) cells during osteoclast differentiation. (**B**) Effect of *Pydc3* RNAi on osteoclast differentiation. Cells stained for TRAP were shown in the left panel. The frequency of TRAP^+^ multinucleated cells was shown in the right panel. (**C**) Effect of overexpression of *Pydc3* on osteoclast differentiation. Expression levels of exogeneous *Pydc3* were confirmed by Western blotting with an anti-Flag antibody and shown in the left panel. Cells stained for TRAP were shown in the middle panel, and the frequency of TRAP^+^ multinucleated cells were shown in the right panel. Data are shown as the mean ± S.D. Each dot represents a technical replicate. *** *p* < 0.001, **** *p* < 0.0001 and NS– not significant. Scale bars represent 100 μm.

We first examined the expression dynamics of *Pydc3* in Pchd7^+/+^ and Pcdh7^−/−^ cells during RANKL-induced osteoclast differentiation. *Pydc3* expression was barely detectable in Pcdh7^+/+^ cells, both in BMMs and during RANKL stimulation (Figure 2A). In contrast, *Pydc3* was highly expressed in Pcdh7^−/−^ BMMs and decreased following RANKL stimulation (Figure 2A). These findings were consistent with the expression profiles observed in the differential gene expression analysis (Figure 1). To assess the functional role of Pydc3, we performed RNAi-mediated gene silencing. Pcdh7^+/+^ and Pcdh7^−/−^ BMMs were transduced with a retrovirus encoding Pydc3-specific shRNA, followed by RANKL stimulation to induce osteoclast differentiation. Knockdown of *Pydc3* significantly restored the impaired osteoclast differentiation observed in Pcdh7^−/−^ cultures, but it had no effect on Pcdh7^+/+^ cells (Figure 2B). We next performed retrovirus-mediated overexpression of *Pydc3* in both Pcdh7^+/+^ and Pcdh7^−/−^ cells. Overexpression of *Pydc3* in Pcdh7^+/+^ cells significantly inhibited osteoclast differentiation to a similar extent to Pcdh7^−/−^ cells (Figure 2C). In contrast, overexpression of Pydc3 in Pcdh7^−/−^ cells had no effect (Figure 2C). These results suggest that *Pydc3* acts as a negative regulator of osteoclast differentiation and that the impaired osteoclast differentiation observed in Pcdh7 deficiency is attributed to Pydc3 upregulation.

### 3.3. Pcdh7 Deficiency Leads to Increased IFN-β Production

**Figure 3:**
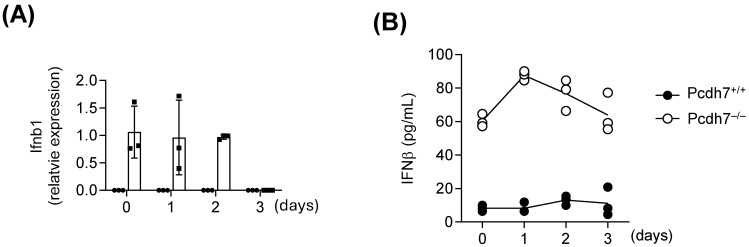
Pcdh7 deficiency resulted in increased levels of both the mRNA and protein of IFN-β. (**A**) Gene expression of *Ifnb* in Pcdh7^+/+^ (circle) and Pcdh7^−/−^ (square) cells during osteoclast differentiation. Data are means ± SD. (**B**) Production of IFN-β in Pcdh7^+/+^ and Pcdh7^−/−^ cells during osteoclast differentiation. The graphs are representative of three independent experiments. Data are means. Each dot represents a technical replicate.

Gene Ontology (GO) enrichment analysis of RNA-sequencing data revealed elevated expression of genes involved in interferon response pathways, including interferon-stimulated genes (ISGs), in Pcdh7^−/−^ cells. Significant enrichment was observed for terms such as “defense response to virus” (GO: 0051607) and “cellular response to interferon-alpha” (GO: 0035457) (Table 1). Additionally, Pydc3 expression has been reported to be induced by IFN-β [14], and human POP3 is known to be involved in type I interferon responses [12]. These findings suggest a potential role for Pcdh7 in regulating type I IFN signaling. To investigate this, we measured both mRNA and protein levels of IFN-β in Pcdh7^+/+^ and Pcdh7^−/−^ cells. BMMs from Pcdh7^+/+^ and Pcdh7^−/−^ mice were cultured with RANKL, and total RNA and culture supernatants were collected daily over a three-day period. *Ifnb* mRNA levels were quantified by qPCR, and IFN-β protein levels were measured by ELISA. Pcdh7^−/−^ BMMs exhibited significantly elevated and sustained *Ifnb* mRNA for two days following RANKL stimulation (Figure 3A). Consistently, increased levels of IFN-β protein were detected in the culture supernatants of Pcdh7^−/−^ cells, both in unstimulated BMMs and following RANKL treatment (Figure 3B). These results indicate that Pcdh7 deficiency leads to increased IFN-β production.

### 3.4. Impaired Osteoclast Differentiation in Pcdh7-Deficient Cells Is Rescued by IFN-β Neutralization

**Figure 4:**
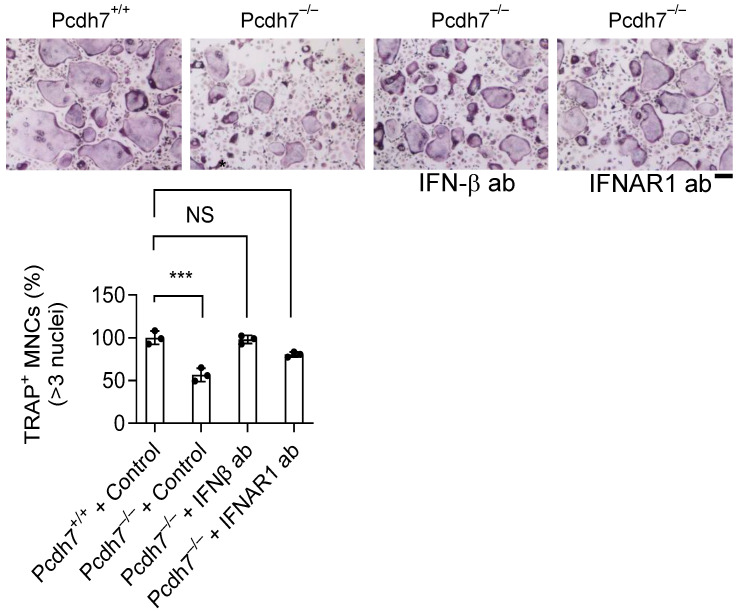
Neutralization of IFN-β signaling restored osteoclast differentiation in Pcdh7 deficiency. Cells stained for TRAP were shown in the top panel. The frequency of TRAP^+^ multinucleated cells was shown in the bottom panel. IFN-β ab: anti-IFN-β antibody, IFNAR1 ab: anti-IFNAR1 antibody. Data are shown as the mean ± S.D. Each dot represents a technical replicate. *** *p* < 0.001 and NS—not significant. The scale bar represents 100 μm.

Given that IFN-β is known to suppress osteoclastogenesis [7], we next tested whether the impaired osteoclast differentiation observed in Pcdh7^−/−^ cells could be reversed by neutralizing IFN-β signaling. Pcdh7^−/−^ BMMs were cultured with RANKL in the presence of neutralizing antibodies against IFN-β or INFAR1 for three days. Pcdh7^+/+^ cells were used as controls. Treatment with either neutralizing antibody significantly restored osteoclast differentiation in Pcdh7^−/−^ cultures, reaching levels comparable to those Pcdh7^+/+^ controls (Figure 4). These results suggest that excessive IFN-β production contributes to the defective osteoclast differentiation associated with Pcdh7 deficiency. Taken together, our findings uncover a novel role for Pcdh7 in regulating IFN-β signaling in osteoclasts.

## 4. Discussion

**Figure 5:**
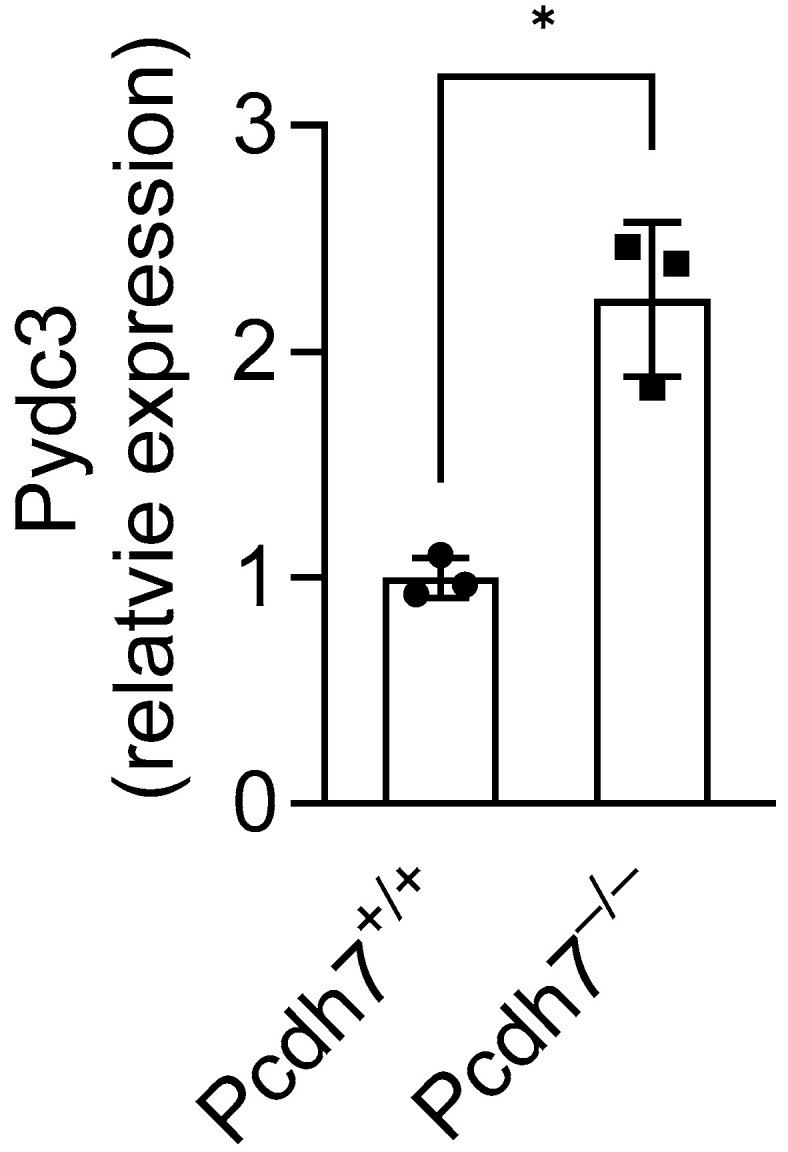
Gene expression of *Pydc3* in Pcdh7^+/+^ and Pcdh7^−/−^ mouse bone tissues. Relative *Pydc3* expression was measured in tibial bone from Pcd7^+/+^ and Pcdh7^−/−^ female mice by qPCR. * *p* < 0.05. Values are presented as mean ± SD, and each dot represents a technical replicate.

In this study, we investigated the gene regulatory role of Pcdh7 and identified Pydc3 as a novel mediator that negatively regulates osteoclast differentiation. Consistent with the results obtained from in vitro experiments, we found significant upregulation of *Pydc3* expression in Pcdh7^−/−^ mouse bone tissues (Figure 5), where osteoclasts and their precursors reside, supporting the critical role of Pcdh7 in the regulation of Pydc3 expression in vivo. We also found that Pcdh7 is involved in IFN-β signaling. However, the trigger for *Pydc3* expression in Pcdh7^−/−^ cells remains unclear. The elevated production of IFN-β in Pcdh7^−/−^ cells likely contributes to the upregulation of *Pydc3*, as both *Pydc3* and its human analog POP3 are known to be induced by IFN-β [12,14]. In turn, *Pydc3* may also enhance IFN-β production, as previously reported [12,14]. These observations suggest that Pydc3 functions as a positive feedback regulator of IFN-β. This feedback loop—in which IFN-β induces *Pydc3* expression, and elevated *Pydc3* further amplifies IFN-β production—ultimately inhibits osteoclast differentiation. Indeed, neutralization of IFN-β signaling using anti-IFN-β or anti-IFNAR1 antibodies restored osteoclast differentiation in Pcdh7^−/−^ cells. Similarly, RNAi-mediated knockdown of Pydc3 fully restored the impaired osteoclast differentiation in Pcdh7-deficient cultures. Osteoclasts are positively and negatively regulated through autocrine factors such as Osteopontin, SFRP4 and IFN-β [7,17,18,19]. Our study revealed a role for Pcdh7 and Pydc3 in the regulation of IFN-β, highlighting the interplay between adhesion molecules and innate immune signaling in the regulation of autocrine-mediated osteoclast differentiation. RANKL stimulation attenuated Pydc3 mRNA expression in Pcdh7^−/−^ BMMs, suggesting that RANKL signaling may negatively regulate Pydc3 expression. Further studies are needed to elucidate the upstream regulatory mechanisms that control *Pydc3* transcription during osteoclast differentiation.

Human POP3 has been shown to inhibit ALR inflammasome activation and regulate the production of inflammatory cytokines such as IL-1 [12]. Pydc3 has also been implicated in the regulation of virus-mediated IL-1β production [14]. In this study, we focused on the role of Pydc3 in regulating IFN-β, as our RNA-seq analysis pointed to type I IFN involvement in the context of Pcdh7 deficiency. However, we did not directly address the potential role of Pydc3 in inflammasome activation. Given that inflammation can promote osteoclast differentiation [20,21,22], it is possible that Pydc3 may also influence this process through inflammasome-related mechanisms. Future studies will be required to determine whether Pydc3-mediated inflammasome activity contributes to the downstream effects of Pcdh7 deficiency.

We previously demonstrated that Pcdh7 functions as a signal transducer: Pcdh7 ligation activates PP2A, which subsequently activates GSK3β, leading to activation of small GTPases such as RhoA, and ultimately promoting osteoclast differentiation and multinucleation [4,15,16]. Indeed, the impaired osteoclast differentiation in Pcdh7^−/−^ cells was fully rescued by overexpression of constitutive active forms of RhoA and Rac1 [15]. In the present study, we similarly showed that osteoclast differentiation in Pcdh7^−/−^ cells was fully restored by Pydc3 knockdown and/or IFN-β signaling neutralization. This raises an important mechanistic question: Is there a link between small GTPases signaling and IFN-β? One study reported that the absence of IFN-β signaling enhanced RhoA activation [23], while another showed that IFN-β signaling inhibited Rac1 activation via suppressor of cytokine signaling 1 (SOCS1) [24]. These findings suggest that IFN-β may negatively regulate the activation of small GTPases. Therefore, neutralization of IFN-β signaling could relieve this inhibition and promote GTPase activity, contributing to the rescue of osteoclast differentiation in Pcdh7-deficient cells. Further investigation is required to determine whether and how Pcdh7 coordinates IFN-β signaling and small GTPase activity to regulate osteoclast differentiation.

Taken together, our findings reveal a previously unrecognized role for Pcdh7 in regulating IFN-β signaling. Specifically, we demonstrate that Pcdh7 suppresses *Pydc3* expression and IFN-β production, thereby facilitating proper osteoclast differentiation.

## Figures and Tables

**Table 1:** Biological processes that were enriched for genes differentially expressed.

GO ID	GO Term	Significant Gene Count	%Significant Genes	Padj-Value	Genes
GO:0051607	defense response to virus	5	3.6	0.0012	*Ifit2*; *Ifit3*; *Ifit3b*; *Ifitm1*; *Itgax*
GO:0035457	cellular response to interferon-alpha	2	22.2	0.018	*Ifit2*; *Ifit3*
GO:0001503	ossification	3	3.3	0.0373	*Ifitm1*; *Mmp9*; *Spp1*
GO:0045780	positive regulation of bone resorption	2	10	0.0373	*Car2*; *Spp1*
GO:0006885	regulation of pH	2	8	0.0451	*Car2*; *Slc9a9*

## Data Availability

Data are contained within the article.

## Supplementary Materials

The following supporting information can be downloaded at: https://www.mdpi.com/article/10.3390/cells14161298/s1, Table S1: Significantly differentially expressed genes.

## References

[1] Teitelbaum S.L. (2000). Bone Resorption by Osteoclasts. Science.

[2] Takegahara N., Kim H., Choi Y. (2024). Unraveling the intricacies of osteoclast differentiation and maturation: Insight into novel therapeutic strategies for bone-destructive diseases. Exp. Mol. Med..

[3] Hayashi S., Takeichi M. (2015). Emerging roles of protocadherins: From self-avoidance to enhancement of motility. J. Cell Sci..

[4] Kim H., Takegahara N., Choi Y. (2023). PP2A-Mediated GSK3β Dephosphorylation Is Required for Protocadherin-7-Dependent Regulation of Small GTPase RhoA in Osteoclasts. Cells.

[5] de Weerd N.A., Nguyen T. (2012). The interferons and their receptors--distribution and regulation. Immunol. Cell Biol..

[6] Chen K., Liu J., Cao X. (2017). Regulation of type I interferon signaling in immunity and inflammation: A comprehensive review. J. Autoimmun..

[7] Takayanagi H., Sato K., Takaoka A., Taniguchi T. (2005). Interplay between interferon and other cytokine systems in bone metabolism. Immunol. Rev..

[8] Takayanagi H., Kim S., Matsuo K., Suzuki H., Suzuki T., Sato K., Yokochi T., Oda H., Nakamura K., Ida N. (2002). RANKL maintains bone homeostasis through c-Fos-dependent induction of interferon-β. Nature.

[9] Place D.E., Malireddi R.K.S., Kim J., Vogel P., Yamamoto M., Kanneganti T.D. (2021). Osteoclast fusion and bone loss are restricted by interferon inducible guanylate binding proteins. Nat. Commun..

[10] Broz P., Dixit V.M. (2016). Inflammasomes: Mechanism of assembly, regulation and signalling. Nat. Rev. Immunol..

[11] Dorfleutner A., Chu L., Stehlik C. (2015). Inhibiting the inflammasome: One domain at a time. Immunol. Rev..

[12] Khare S., Ratsimandresy R.A., de Almeida L., Cuda C.M., Rellick S.L., Misharin A.V., Wallin M.C., Gangopadhyay A., Forte E., Gottwein E. (2014). The PYRIN domain-only protein POP3 inhibits ALR inflammasomes and regulates responses to infection with DNA viruses. Nat. Immunol..

[13] Brunette R.L., Young J.M., Whitley D.G., Brodsky I.E., Malik H.S., Stetson D.B. (2012). Extensive evolutionary and functional diversity among mammalian AIM2-like receptors. J. Exp. Med..

[14] Vijay R., Fehr A.R., Janowski A.M., Athmer J., Wheeler D.L., Grunewald M., Sompallae R., Kurup S.P., Meyerholz D.K., Sutterwala F.S. (2017). Virus-induced inflammasome activation is suppressed by prostaglandin D(2)/DP1 signaling. Proc. Natl. Acad. Sci. USA.

[15] Kim H., Takegahara N., Choi Y. (2021). Protocadherin-7 Regulates Osteoclast Differentiation through Intracellular SET-Binding Domain-Mediated RhoA and Rac1 Activation. Int. J. Mol. Sci..

[16] Kim H., Takegahara N., Walsh M.C., Ueda J., Fujihara Y., Ikawa M., Choi Y. (2020). Protocadherin-7 contributes to maintenance of bone homeostasis through regulation of osteoclast multinucleation. BMB Rep..

[17] Chen K., Ng P.Y., Chen R., Hu D., Berry S., Baron R., Gori F. (2019). Sfrp4 repression of the Ror2/Jnk cascade in osteoclasts protects cortical bone from excessive endosteal resorption. Proc. Natl. Acad. Sci. USA.

[18] Yao Z., Xing L., Qin C., Schwarz E.M., Boyce B.F. (2008). Osteoclast precursor interaction with bone matrix induces osteoclast formation directly by an interleukin-1-mediated autocrine mechanism. J. Biol. Chem..

[19] Chellaiah M.A., Kizer N., Biswas R., Alvarez U., Strauss-Schoenberger J., Rifas L., Rittling S.R., Denhardt D.T., Hruska K.A. (2003). Osteopontin deficiency produces osteoclast dysfunction due to reduced CD44 surface expression. Mol. Biol. Cell.

[20] Terkawi M.A., Matsumae G., Shimizu T., Takahashi D., Kadoya K., Iwasaki N. (2022). Interplay between Inflammation and Pathological Bone Resorption: Insights into Recent Mechanisms and Pathways in Related Diseases for Future Perspectives. Int. J. Mol. Sci..

[21] Epsley S., Tadros S., Farid A., Kargilis D., Mehta S., Rajapakse C.S. (2020). The Effect of Inflammation on Bone. Front. Physiol..

[22] Amarasekara D.S., Yun H., Kim S., Lee N., Kim H., Rho J. (2018). Regulation of Osteoclast Differentiation by Cytokine Networks. Immune Netw..

[23] Daniels B.P., Holman D.W., Cruz-Orengo L., Jujjavarapu H., Durrant D.M., Klein R.S. (2014). Viral pathogen-associated molecular patterns regulate blood-brain barrier integrity via competing innate cytokine signals. mBio.

[24] Inoue M., Williams K., Oliver T., Vandenabeele P., Rajan J.V., Miao E.A., Shinohara M.L. (2012). Interferon-b Therapy Against EAE Is Effective Only When Development of the Disease Depends on the NLRP3 Inflammasome. Sci. Signal..

